# Relationship between cell ploidy and glucocorticoid induced death in human lymphoid cell lines.

**DOI:** 10.1038/bjc.1984.115

**Published:** 1984-06

**Authors:** J. E. Dyson, P. Quirke, C. C. Bird, J. B. McLaughlin, C. R. Surrey

## Abstract

**Images:**


*Br. J. Cancer* (1984), **49**, 731–738

### Relationship between cell ploidy and glucocorticoid induced death in human lymphoid cell lines

J.E.D. Dyson[1], P. Quirke[2], C.C. Bird[2], J.B. McLaughlin[1] & C.R. Surrey[1]

[1]*University Department of Radiotherapy, Tunbridge Building, Cookridge Hospital, Leeds LS16 6QB, and* [2]*Department of Pathology, University of Leeds, Leeds LS2 9JT, UK.*

**Summary** We have found a relationship between sensitivity to glucocorticoid induced cell death (at 10 $\mu$M glucocorticoid) and ploidy in the human lymphoid cell line CCRF/CEM-C7. Most sensitive clones are diploid, whilst resistant clones and the resistant parent line CCRF/CEM are tetraploid. Diploid sensitive clones have a tendency to become aneuploid within a few months of isolation, with alterations in their kinetic responses to glucocorticoids. This is followed by a doubling in DNA content which results in reversion to the tetraploid glucocorticoid resistant state of the parent line CCRF/CEM. A few sensitive clones have been found to be tetraploid but with different kinetic responses to glucocorticoids as compared to diploid clones. The principal difference being an extended lag period (48–72 h) prior to lethal response. The relationship between ploidy and glucocorticoid sensitivity does not appear to extend to other human lymphoid cell lines.

The advent of flow cytofluorometry has presented investigators with a rapid and accurate method of establishing DNA content profiles for cell populations and mixtures of populations. With appropriate staining techniques this method is also capable of resolving small differences in ploidy.

We have been using this technique to investigate the phenomenon of programmed cell death, now generally referred to as apoptosis (Kerr *et al.*, 1972), employing as a model glucocorticoid induced cell death in human lymphoid cell lines (HLCL). For this purpose both glucocorticoid sensitive (lethal concentration 1 to 10 $\mu$M) and insensitive (lethal concentration 100 to 1,000 $\mu$M) HLCL have been employed. Changes in DNA content profile in the presence of glucocorticoid were monitored over a period of 3 to 14 days and correlated with lethal responses measured by other physical techniques.

During the course of these studies heterogeneity in sensitivity of cultures to glucocortocoid treatment made the kinetics of the lethal responses difficult to interpret. This appeared to relate to the heterogeneity of the cultures themselves, as all were found to contain from 2 to 5 cell sub-populations of different ploidy after periods of several weeks to a few months of continuous culture. Various changes in ploidy occurring during measurements also suggested that with certain HLCL a strong correlation exists between increased resistance to glucocorticoid induced cell death and increase in DNA content of the cells. This association was found to hold both for spontaneous and experimentally induced resistance to glucocorticoid treatment.

Correspondence: J.E.D. Dyson.
Received 5 December 1983; accepted 23 February 1984.

We present our findings as they have considerable implications for those employing HLCL as *in vitro* models of glucocorticoid-induced effects.

### Materials and methods

### *Cell lines*

The origin, age in culture, and karyotypes of the HLCL studied have been previously described (Burrow *et al.*, 1981). C7, C14, C14/7, C15 and C24/2C are clones derived from the glucocorticoid sensitive CCRF/CEM-C7 line (Norman & Thompson, 1977) by limiting dilution. This line was initially derived from the parent glucocorticoid resistant CCRF/CEM line.

### *Cell cultures*

HLCL were grown in suspension culture in Nunclon culture flasks in RPMI 1640 medium (Flow Laboratories Ltd., Irvine, Ayrshire), supplemented with 10% donor calf serum (heat inactivated 1 h at 56°C) (Gibco Ltd., Paisley, Scotland), together with final concentrations of 20 mM 3-(N-morpholino)propane-sulphonic acid (MOPS) (Sigma Chemical Co. Ltd., Poole, Dorset), 4 mM L-glutamine, 3.7 units $ml^{-1}$ streptomycin (Glaxo Laboratories, Greenford) and 5 units $ml^{-1}$ benzylpenicillin (Crystapen, Glaxo). Methylprednisolone sodium succinate (Solu-Medrone, Upjohn Ltd.) (MPSS) dissolved in distilled water was added to experimental cultures to give the desired final concentration. An equal quantity of distilled water was added to the control cultures. All cultures were maintained at 37°C.

For flow cytofluorometry aliquots were removed from experimental and control cultures at desired

© The Macmillan Press Ltd., 1984

intervals, washed × 2 with PBS and finally resuspended in PBS plus 10% Hanks basic salt solution (HBSS). Cells for DNA profile measurement were removed from cultures growing in log phase, washed × 2 in PBS, treated with 0.01% Triton X-100 for 45 sec, washed × 2 in PBS, and finally resuspended in PBS plus 10 mM $MgSO_4$ for mithramycin staining (Crissman & Tobey, 1974).

*Flow cytofluorometry*

*Staining procedures* To determine the percentage of dying and dead cells in each cell aliquot, the following procedure was adopted. To the cell suspension in PBS plus 10% HBSS an equal volume of the same medium was added containing the stains acridine orange (AO) and ethidium bromide (EB) to give final concentrations of 15 μM and 3 μM respectively. Viable cells are only permeable to AO which intercalates double-stranded DNA to give green fluorescence (Darzynkiewicz, 1979). This concentration of AO however in the presence of low concentrations of $Ca^{2+}$ and $Mg^{2+}$ (from the HBSS) denatures RNA to single stranded form, on which it stacks electrostatically to give red fluorescence (Darzynkiewicz, 1979). The cell membranes of dying and dead cells are permeable to EB, which gives a strong red fluorescence with both nucleic acids (Fischer & Gill, 1977). Green fluorescence from AO, which enters with the EB, is partially or wholly suppressed by energy transfer between the two stains (Stöhr & Vogt-Schaden, 1979). Computer analysis by directly outlining the population of viable cells on the scattergram (see Figure 1) allows determination of the percentage of viable cells in the cell aliquot.

Comparison of results obtained by this procedure with more conventional methods of counting nigrosine stained cells in a haemocytometer, showed that the two methods are entirely comparable. The flow cytofluorometric method is subject to less uncertainty as to whether a cell is stained or not, since the extent of EB entry into the cell is directly measurable.

For high resolution DNA profiles mithramycin staining was employed (Crissman & Tobey, 1974). For this purpose an equal volume of PBS plus 10 mM $MgSO_4$ containing the mithramycin (Sigma) was added to give a final concentration of 5 μM of the stain.

For both staining methods the number of cells was adjusted so as not to exceed $5 \times 10^5$/2 ml of suspension.

*Measurement* All flow cytofluorometric measurements were carried out with an Ortho-Diagnostic Systems Cytofluorograf Systems 50H with a Lexel 95-4w argon ion laser routinely used at 250 mW at the 488 nm line. With the dichroic mirror and filter systems employed the wavelengths measured were: green fluorescence 530–565 nm and red flourescence >640 nm. The Cytofluorograf is interfaced to an Ortho 2151 computer system.

With AO plus EB staining the cell aliquot was placed in the flow cytometer after 27 min, sample

**Figure 1** Flow cytometric scattergrams of clone C14 cells, stained with acridine orange plus ethidium bromide, (a) control cells 96 h; (b) cells incubated 96 h with 10 μM MPSS. Ploidy of 4c indicated in this figure, together with percentages of viable and dying plus dead cells determined by computer graphical analysis of areas shown. Cell number represented by grey scale intensity.

flow started and allowed to equilibrate. After a further 3 min had elapsed data acquisition into computer memory was commenced. This ensured reproducibility of the period of time during which entry of EB into cells could take place.

With mithramycin staining measurements were carried out 15 min after addition of stain.

Calibration of the fluorescence intensity corresponding to a diploid (2c) DNA content was carried out with normal human lymphocytes. These were isolated from peripheral blood samples by layering on to lymphocyte separation medium (Flow Laboratories, Irvine, Ayrshire). For calibration purposes the lymphocytes were stained in an identical manner to the cells being measured.

### Results

Representative DNA content profiles of various cultures of CCRF/CEM-C7 and of CCRF/CEM are depicted in Figure 2. The major and minor peaks of the DNA profiles of the other HLCL studied are presented in Table I. The parent glucocorticoid resistant line CCRF/CEM shows a predominantly tetraploid profile (Figure 2D). With the exception of C14 and C14/7 (Table I) glucocorticoid sensitive clones derived from the sensitive cell line CCRF/CEM-C7 show essentially diploid profiles. The DNA profile of a diploid clone determined within a few weeks of isolation is represented by culture C7A (Figure 2A). Culture C7B was derived from the same recloning as culture C7A, but, after several months in culture, had become aneuploid, with a principal peak at 2.4C (Figure 2B). Culture C7C was also derived from the same recloning as C7A, but was regrown from cells stored in liquid nitrogen for several months. Possibly due to the shorter period in continuous culture C7C showed at this time less tendency to aneuploidy than C7B (Figure 2C). Culture $C7_{reverted}$ ($C7_{rev}$) was derived by spontaneous reversion of culture C7B to the resistant state, associated with development of a tetraploid profile comparable to that of the parent resistant line CCRF/CEM (Figure 2, Table I). Both C7A and C7C also subsequently reverted to resistance/tetraploidy in an analogous manner to C7B. Culture C7A, however, maintained an approximately diploid DNA profile for several months longer, in continuous culture, than culture C7B.

**Figure 2** Cell DNA content profiles for various cultures of clone C7 of the glucocorticoid susceptible cell line CCRF/CEM-C7, and of the resistant parent line CCRF/CEM. Mithramycin staining as described in the text. Calibrated for diploid DNA (2c) content at channel 300 as described in the text.

Culture $C24_{rev}$ was obtained during incubation of a culture of C24/2C with 10 $\mu$M of MPSS (Table I). Steroid sensitive cells were killed by treatment and the resistant cells which overgrew the culture showed a tetraploid profile similar to CCRF/CEM (Figure 2D). Comparison of the DNA profile of a culture of clone C24/2C determined on 19th August (Table I) and on 11th October (Table I) suggested this culture also was reverting spontaneously to the resistant state, confirmed by measurement on 7th December when this culture was found to be resistant/tetraploid (Table I).

Plots of cell viability as a function of time of incubation with 10 $\mu$M MPSS are depicted in Figure 3 for glucocorticoid sensitive clones. It is apparent that C7A, C7B, C7C and C24/2C (Figure 3) were all sensitive to the action of 10 $\mu$M MPSS, with 70% cells killed by 72 h. The truly diploid culture C7A was most glucocorticoid sensitive. With increased aneuploidy (cultures C7B and C7C) the rate at which cell death occurs appears to decrease, although there is no alteration in lag period (Figure 3). Clone C15 on the other hand was much less sensitive to 10 $\mu$M MPSS with only 20% cells responding lethally by 72 h (Figure 3). In addition to a diploid component it contained a large tetraploid component (Table I). Clones C14 and C14/7 (C14/7 was recloned from C14) are exceptions to the relationship between the diploid state and sensitivity. Both are clearly tetraploid (Table I) yet sensitive to 10 $\mu$M MPSS (Figure 3); both are unusual, however, in showing a 48 h lag period before responding to MPSS, in contrast to

**Table I** Summary of peaks in DNA content profiles of the cell types studied[a] and response to glucocorticoid treatment.[b]

| *Cell-line* | *Culture* | *Response to glucocorticoid*[b] | *2.0c* | *DNA peaks at:* *2.4c* | *3.6c* | *4.0c* | *4.8c* |
|---|---|---|---|---|---|---|---|
| CCRF/CEM | — | resistant | minor | minor | major | major | major |
| CCRF/CEM-7 | C7A | sensitive | major | — | — | — | — |
| CCRF/CEM-7 | C7B | sensitive | — | major | — | minor | — |
| CCRF/CEM-7 | C7C | sensitive | major | major | — | — | minor |
| CCRF/CEM-7 | C24/2C at 19/8 | sensitive | major | shoulder | minor | — | — |
| CCRF/CEM-7<br>CCRF/CEM-7 | C/24/2C<br>at 11/10 | sensitive | major | major | minor | — | — |
| CCRF/CEM-7 | C24/2C at 7/12 | resistant | minor | minor | major | major | major |
| CCRF/CEM-7 | $C7_{rev}$ | resistant | minor | minor | major | major | major |
| CCRF/CEM-7 | $C24_{rev}$ | resistant | minor | minor | major | major | major |
| CCRF/CEM-7 | C15 | partially sensitive | major | — | — | minor | — |
| CCRF/CEM-7 | C14 | sensitive | — | — | — | major | — |
| CCRF/CEM-7 | C14/7 | sensitive | — | — | — | major | — |
| F-89 | — | resistant | — | — | — | major | — |
| Molt-4-F | — | resistant | — | major | major | — | — |
| $EB_1$ | — | resistant | — | major | — | — | minor |
| $EB_23945$ | — | resistant | major | — | — | minor | — |

[a]Determined as described in the text.
[b]Response to 10 $\mu$M MPSS as described in the text.

**Figure 3** Cell viability as a function of time of incubations with 10 $\mu$M MPSS for sensitive clones of CCRF/CEM-C7. Viability determined as described in text.

the 24 h lag period of other sensitive clones. Both these clones have been monitored in continuous culture for a period of some 6 months. Neither has shown any tendency to alterations in DNA content profile, or in glucocorticoid sensitivity.

Clones $C7_{rev}$, $C24_{rev}$ and the parent cell line CCRF/CEM, were all approximately tetraploid (Figure 2, Table I), although some diploid cells were present. All were resistant to 10 $\mu$M MPSS for 7 to 10 days (Table I) although we observed a small loss of viability with continued exposure to this concentration of steroid after this time. Cell line F89 was clearly tetraploid (Table I) and was resistant to 10 $\mu$M MPSS. Cell line Molt-4-F was however near diploid (2.4c) although with a tetraploid component (Table I), but was very resistant to MPSS. After 14 days at 1,000 $\mu$M MPSS this particular strain of Molt-4-F still showed nearly 60% viability, whereas other resistant lines responded lethally to this suprapharmacological concentration of hormone in a much shorter period of time. Cell line $EB_1$ was 2.4c with an additional component of 4.8c (Table I). $EB_2$-3945 was diploid with a considerable content of 4c cells (Table I). Both cell lines were very resistant to MPSS, showing >80% viable cells at 72 h in the presence of 1,000 $\mu$M MPSS, with ~15% viable cells still present at 7 days. In the presence of 10 $\mu$M MPSS both $EB_1$ and $EB_2$-3945 showed >95% viable cells at 7 days.

DNA content profiles determined at 24 h intervals during incubation of the glucocorticoid sensitive clone (C24/2C with 10 $\mu$M MPSS are depicted in Figure 4. There was a transient increase in the apparent DNA content of the 2c $G_1$ peak (at channel 330, Figure 4b), and an accumulation of hypertetraploid cells (channels >600) (Figure 4b) due, apparently, to inhibition of division of cells in $G_2$ (at channel 600, Figure 7a). This appears to correspond to the 24 h lag period generally observed with glucocorticoid induced cell death. Subsequent to this (48 h, Figure 4c) cells began to accumulate in $G_1$ and die (cells in channels <250 are dying or dead). After 72 h incubation with steroid viability had decreased to 15%, although a few hypertetraploid cells began to appear (Figure 4d). By 96 h incubation re-growth of glucocorticoid resistant cells commenced and viability increased to 49% with the $G_1$ peak now being approximately tetraploid (channel 550, Figure 4e). The final result of this selection process for cells resistant to glucocorticoid was analogous to the parent cell line CCRF/CEM (Figure 2).

A series of DNA profiles were determined at intervals during spontaneous reversion of culture C7B to the resistant state (Figure 5). This occurred over a period of 42 days. The initial culture was approximately diploid although aneuploidy was

**Figure 4** Cell DNA content profiles determined at 24 h intervals during incubation of clone C24/2C with 10 $\mu$M MPSS. (a) DNA profile of control culture without addition of MPSS. Calibration for DNA content as Figure 2.

already apparent (16th May—Figure 5a), but by the end of the period (27th June—Figure 5d), was essentially tetraploid, although a small diploid component remained. Response to incubation with 10 $\mu$M MPSS is shown in Figure 5e. On the 16th May, when the culture was near diploid, this resulted in almost total loss of viable cells after 72 h. On 27th June with reversion to the tetraploid state, the culture was unaffected by 72 h incubation with 10 $\mu$M MPSS. On intermediate dates (6th June, Figure 5b and 17th June, Figure 5c) there was a mixture of near diploid and tetraploid cells. The dashed lines in Figures 5b and 5c indicate DNA profiles for cells incubated on those dates with 10 $\mu$M MPSS for 48 h. Clearly the major effect of glucocorticoid is on near diploid cells, whilst the growth of tetraploid cells is apparently enhanced (Figures 5b and c). This explains why little loss in cell viability occurred following incubation with 10 $\mu$M MPSS on those dates. The DNA profile of the culture depicted in Figure 5d after maintenance

**Figure 5** Cell DNA content profiles determined at intervals from 16th May to 27th June, during spontaneous reversion of culture C7B of CCRF/CEM-C7 to the resistant strain. (b) and (c) Broken lines denote DNA content profiles determined after 48 h incubation with 10 $\mu$M MPSS. Calibration for DNA content as Figure 2. (e) Cell viability as a function of time of incubation with 10 $\mu$M MPSS at dates corresponding to DNA content profiles. Viability determined as described in the text.

in culture for several months was again analogous to that of the parent line CCRF/CEM (Figure 2d).

### Discussion

Examination of the DNA content profiles of various cultures of clones of CCRF/CEM-C7 and of the parent line CCRF/CEM shows that they contain cells of various ploidies 2c, 2.4c, 3.6c, 4c and 4.8c. In general cells of ploidy 2c and 2.4c predominate in the sensitive clones, and cells of higher ploidy in the resistant clones. This is by no means absolute since clone 14 of C7, and reclones from it (clone 14/7), are tetraploid yet sensitive, albeit with differing kinetics from that of the diploid clones. All the diploid clones of CCRF/CEM-C7 show a strong tendency to aneuploidy, and the degree of aneuploidy present is generally related to the period cells have been maintained in continuous culture. The main component of culture C7B for example was 2.4c (Figure 2b) with a considerable tetraploid component. This line had been maintained in culture for much longer than the related culture C7C (regrown from liquid nitrogen) which shows a mixture of ploidies of 2c and 2.4c, although some 4.8c cells are present (Figure 2). The DNA profile of C24/2C measured on 19th August was largely 2c although with a shoulder at 2.4c (Table I). By 11th October this clone revealed equal numbers of cells of 2c and 2.4c together with a 3.6c component (Table I), and by 7th December had reverted altogether to the DNA content profile of the parent cell-line CCRF/CEM (Figure 2).

The resistant cell line, CCRF/CEM, and the reverted strains $C7_{rev}$ and $C24_{rev}$ contain as principal component cells of 3.6c, 4c, and 4.8c, although approximately diploid cells are present as minor components (Figure 2d, Table I).

Aneuploidy develops within a period of a few months of isolation of a diploid susceptible clone of CCRF/CEM-C7. Within a given clone certain cultures (C7A) maintain a diploid profile several months longer than others (C7B, C7C). In our experience development of aneuploidy always precedes reversion to tetraploidy. A few weeks to months after development of aneuploidy certain cells in a culture double in DNA content, these cells eventually outgrow the approximately diploid cells resulting in a reversion to the tetraploid state and glucocorticoid resistance of the parent line CCRF/CEM, with principal peaks at 3.6, 4.0 and 4.8c (Figure 2). In some aneuploid cultures glucocorticoid resistant cells are apparently already present since, for example, in C24/2C, the selective advantage given by 10 $\mu$M MPSS treatment resulted in reversion to resistance/tetraploidy within a few days (Figure 4). In cultures of other clones, apparently equally aneuploid, this has not been observed.

Changes in glucocorticoid binding capacity on reversion to the resistant state have not yet been studied. Previous work (Bird *et al.*, 1977; Barret *et al.*, 1981) has shown no general relationship between glucocorticoid sensitivity and glucocorticoid binding capacity. However a comparison of CCRF/CEM and CCRF/CEM-C7 did show an increased binding of prednisolone by C7 but not of dexamethasone (Barrett *et al.,* 1981). Now we are aware of the instability of diploid susceptible clones, changes in glucocorticoid binding together with other parameters will be monitored during reversion to the resistant state.

In a previous investigation (Blewitt *et al.,* 1983) no differences in ultrastructure were discernible between glucocorticoid susceptible and resistant cells, nor were morphological changes occurring during apoptosis different in the two cell types. We are extending these observations but, so far, electron microscopy has revealed no changes in ultrastructure during reversion from the glucocorticoid susceptible to the resistant state (see also Robertson *et al.,* 1978).

The glucocorticoid susceptible tetraploid clones (C14, C14/7) do not appear unstable, and over a period of 6 months have shown no tendency to aneuploidy or changes in glucocorticoid sensitivity. The other cell lines studied appear less prone to aneuploidy (Table I), and, when aneuploidy is present this does not lead to instability in the DNA content profiles.

Several workers have employed HLCL to study the mechanisms of glucocorticoid-induced cell death in human lymphoid cells (Bird *et al.,* 1975, 1977; Norman & Thompson 1977; Robertson *et al.,* 1978; Burrow *et al.,* 1981; Barrett *et al.,* 1981; see also Munck & Crabtree, 1981). One of the problems encountered in such studies has been the lack of synchronization in response of cells to glucocorticoid treatment. A mandatory lag period of 24–48 h appears to exist before lethal responses commence and these proceed thereafter in an asynchronous fashion (Blewitt *et al.,* 1983). Hitherto, lack of synchrony in response has been attributed to cell kinetic factors, (Harmon *et al.,* 1979). Our results suggest that, in addition to these factors, asynchronous responses may also be due to heterogeneity within the cultures employed. After some months in culture all diploid clones of the sensitive line CCRF/CEM-C7 have been found to be aneuploid and the major component was no longer 2c but 2.4c (cf. Figure 2a to c) and tetraploid cells were present. As some of these cells with increased ploidy appear capable of continued growth in the presence of low concentration of glucocorticoids, and their growth may in fact be enhanced by removal of 2c cells (cf. Figures 4 and 5), the overall kinetics observed may differ from those of the original culture when isolated (cf. C7A, C7B, C7C, Figure 3).

It would thus appear that diploid clones of CCRF/CEM-C7 sensitive to glucocorticoids are inherently unstable, with a tendency to aneuploidy and subsequent reversion to the tetraploid state of the original resistant parent line (CCRF/CEM). As these changes can take place gradually over a period of several months, and with only subtle changes in DNA profile occurring at first, considerable caution needs to be exercised in employing such cultures as *in vitro* models of glucocorticoid induced cell death. There may be advantages in employing more stable clones such as C14 and C14/7 that are tetraploid, yet sensitive to glucocorticoids, although there appears to be differences in the kinetics of their responses compared with diploid clones (compare lag periods C14 and C14/7 with C7A, B, C (Figure 3). Other workers have noted chromosomal gains in HLCL in continuous culture (Steel *et al.,* 1977), and although we measure DNA content profiles rather than karyotypes, it may be that what we see as a tendency to aneuploidy, followed by doubling in DNA content, is equivalent to non-random gains in chromosomal numbers. Such gains may be associated with reversion to the resistant state as in the parent CCRF/CEM line. The steroid susceptible clones of CCRF/CEM that are tetraploid, C14 and C14/7, do not have the aneuploid DNA profiles (3.6c, 4.0c, 4.8c) associated with resistant clones (Table I), so that possibly aneuploidy, rather than tetraploidy, may be associated with resistance to glucocorticoids.

Certain aspects of our results also agree with those of Gledhill *et al.* (1983) regarding acquisition of resistance in tetraploid C7 cells. The tetraploid susceptible clones (C14, C14/7) have remained stable over an observation period of 6 months. In contrast all diploid susceptible clones so far isolated have developed some degree of aneuploidy within 3 months, and reverted to the resistant/tetraploid state within 12 months of continuous culture. This is supported by observations over several years, during which it has been found that glucocorticoid susceptible clones have always lost their susceptibility within approximately 12 months of isolation (when maintained in continuous culture).

This investigation was supported by a grant from the Yorkshire Cancer Research Campaign.

### References

BARRETT, I.D., PANESAR, N.S., BIRD, C.C., ABBOTT, A.C., BURROW, H.M. & STEEL, C.M. (1981). Human lymphoid cell lines and glucocorticods: II. Whole cell and cytoplasmic binding properties of lymphoblastoid, leukaemia and lymphoma lines. *Diagnos. Histopathol.*, **4**, 189.

BIRD, C.C., WADDELL, A.W., ROBERTSON, A.M.G., CURRIE, A.R., STEEL, C.M. & EVANS, J. (1975). Cytoplasmic receptor levels and glucocorticoid responses in human lymphoblastoid cell lines. *Br. J. Cancer*, **32**, 700.

BIRD, C.C., ROBERTSON, A.M.G., READ, J. & CURRIE, A.R. (1977). Cytolethal effects of glucocorticoids in human lymphoblastoid cell lines. *J. Pathol.*, **123**, 145.

BLEWITT, R.W., ABBOTT, A.C. & BIRD, C.C. (1983). Modes of cell death induced in human lymphoid cells by high and low doses of glucocorticoid. *Br. J. Cancer*, **47**, 477.

BURROW, H.M., BIRD, C.C., WARREN, J.V., STEEL, C.M. BARRETT, I.D. & PANESAR, N.S. (1981). Human lymphoid cell lines and glucocorticoids: I. Characterization and cytolethal responses of lymphoblastoid, leukaemia and lymphoma lines. *Diagnos. Histopathol.*, **4**, 175.

CRISSMAN, H.A. & TOBEY, R.A. (1974). Cell cycle analysis in 20 minutes. *Science*, **184**, 1297.

DARZYNKIEWICZ, Z. (1979). Acridine orange as a molecular probe in studies of nucleic acids in situ. In *Flow Cytometry and Sorting*. (Eds. Melamed *et al.*). New York: John Wiley & Sons, p. 285.

FISCHER, C.L. & GILL, C.W. (1977). A cytoflourograf assay of blast transformation. *Ortho Inst. Protocols*, **23**.

GLEDHILL, R.M., GRAY, D.A., SOLBERG-SCOTT, M. & NORMAN, M.R. (1983). Decreased acquisition of glucocorticoid resistance in tetraploid human lymphoid cells. *Mol. Cell. Endocrincol.*, **29**, 67.

HARMON, J.M., NORMAN, M.R., FOWLKES, B.J. & THOMPSON, E.B. (1979). Dexamethasone induces irreversible $G_1'$ arrest and death of a human lymphoid cell line. *J. Cell. Physiol.*, **98**, 267.

KERR, J.F.R., WYLLIE, A.H. & CURRIE, A.R. (1972). Apoptosis: A basic biological phenomenon with wide-ranging implications in tissue kinetics. *Br. J. Cancer*, **26**, 239.

MUNCK, A. & CRABTREE, G.R. (1981). Glucocorticoid induced lymphocyte cell death. In *Cell Death in Biology and Pathology*. (Ed. Bowen & Lockshin). London: Chapman and Hall, p. 329.

NORMAN, M.R. & THOMPSON, E.B. (1977). Characterization of a glucocorticoid-sensitive human lymphoid cell line. *Cancer Res.*, **37**, 3785.

ROBERTSON, A.M.G., BIRD, C.C., WADDELL, A.W. & CURRIE, A.R. (1978). Morphological aspects of glucocorticoid-induced cell death in human lymphoblastoid cells. *J. Path.*, **126**, 181.

STEEL, C.M., WOODWARD, M.A., DAVIDSON, C., PHILIPSON, J & ARTHUR, E. (1977). Non-random chromosome gains in human lymphoblastoid cell lines. *Nature*, **270**, 349.

STÖHR, M. & VOGT-SCHADEN, M. (1979). A new dual staining technique for simultaneous flow cytometric DNA analysis of living and dead cells. In *Flow Cytometry IV*. (Ed. Laerum *et al.*). Bergen: Universitetsforlaget, p. 96.


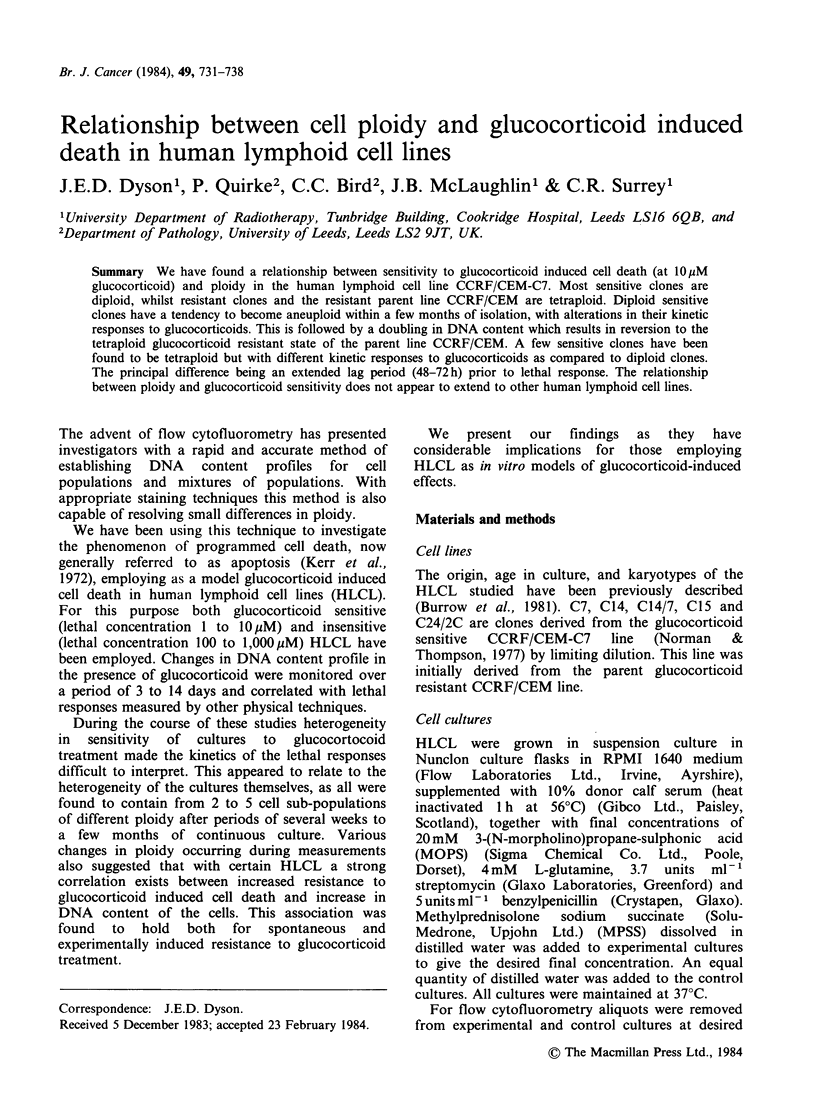

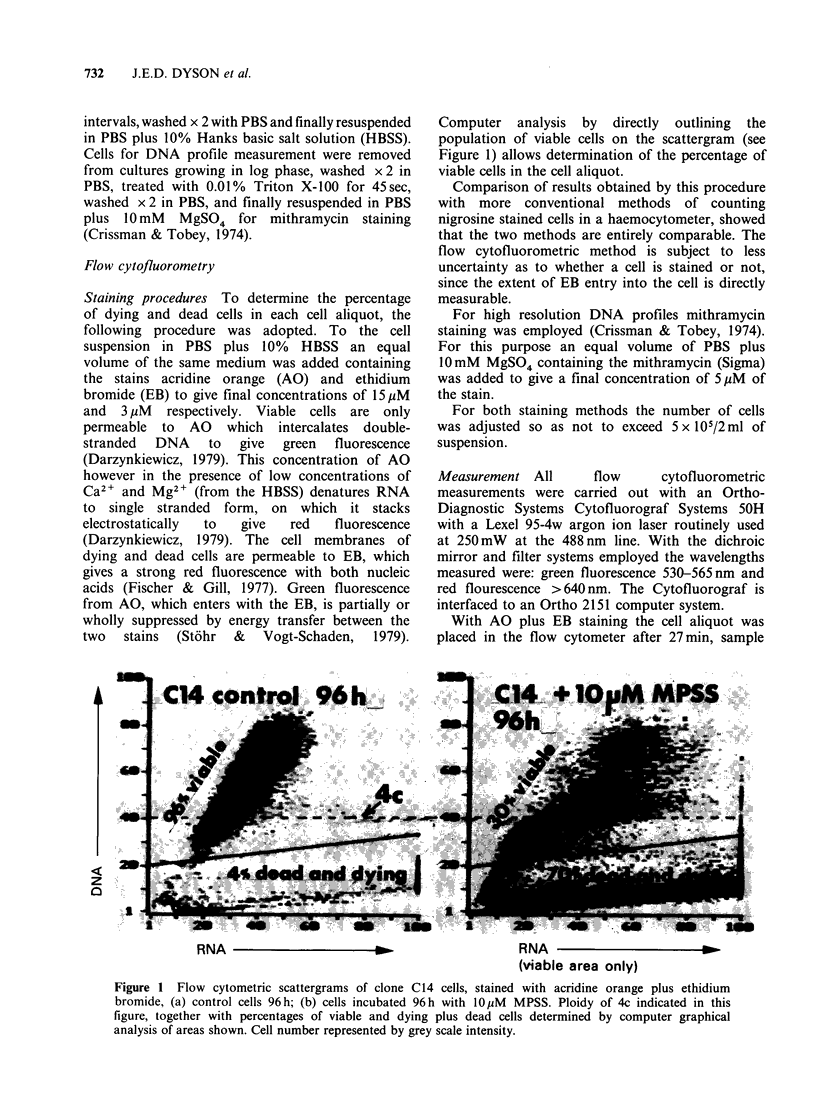

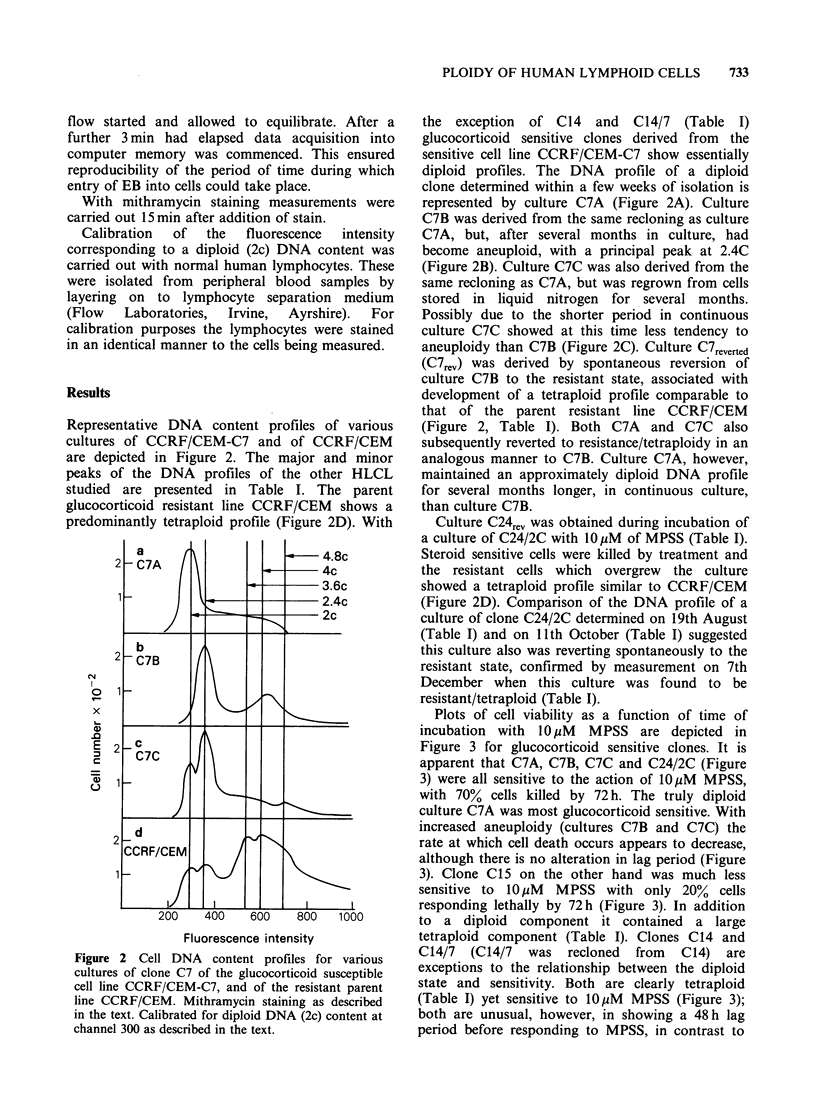

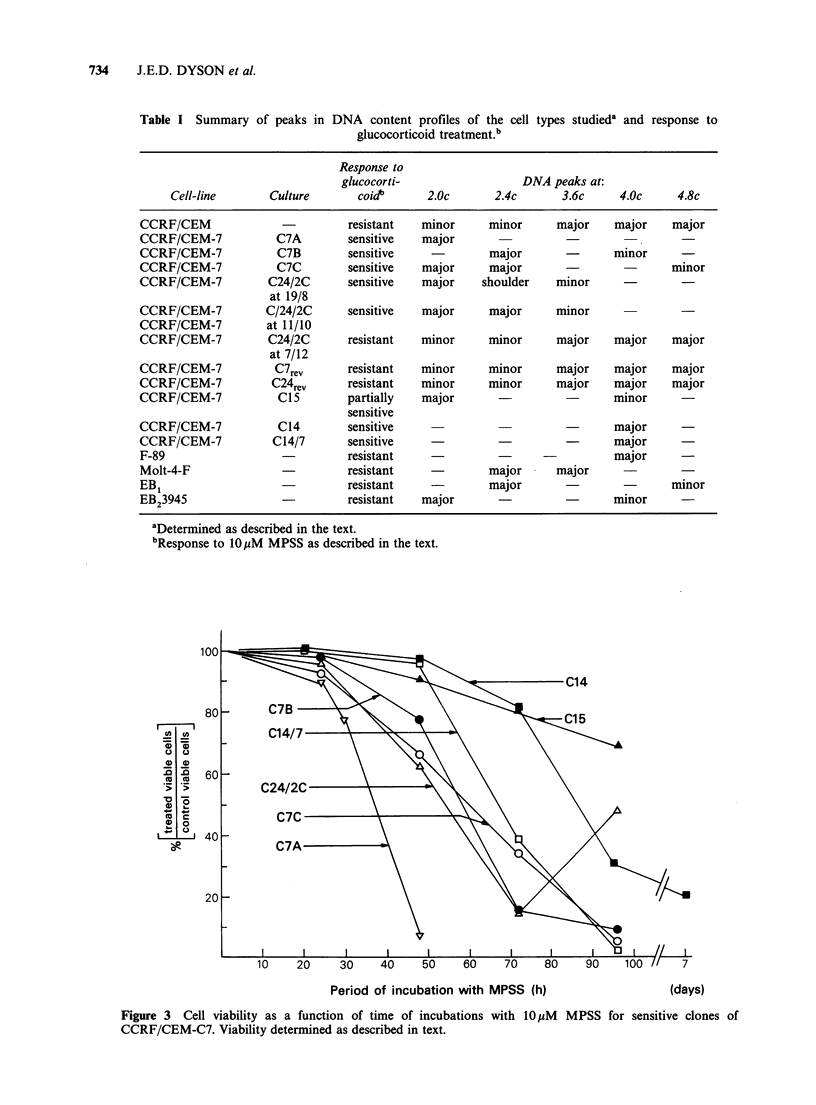

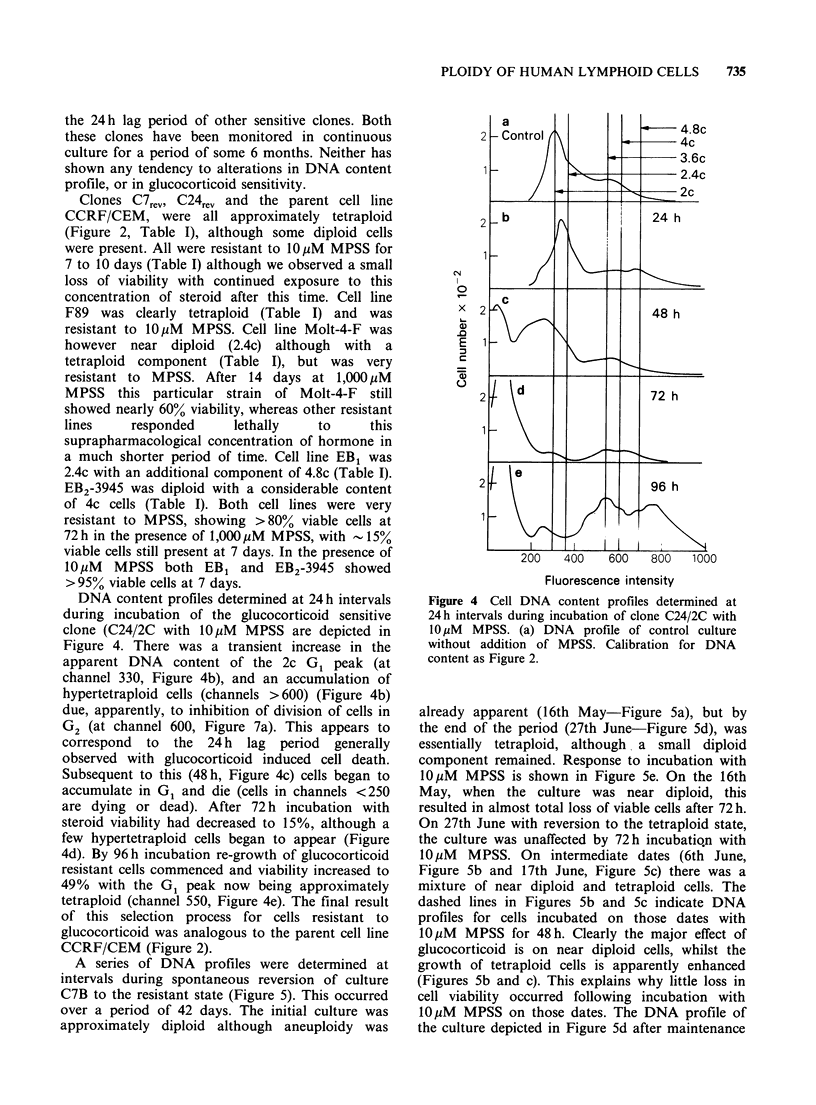

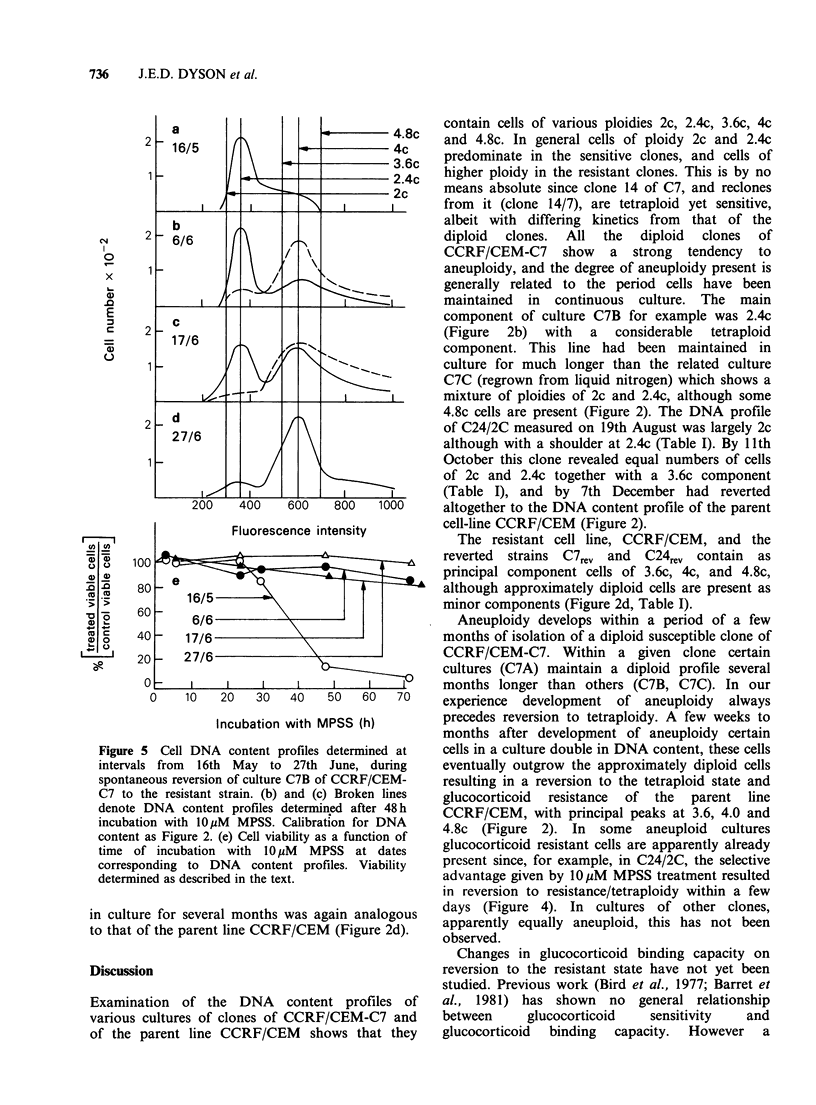

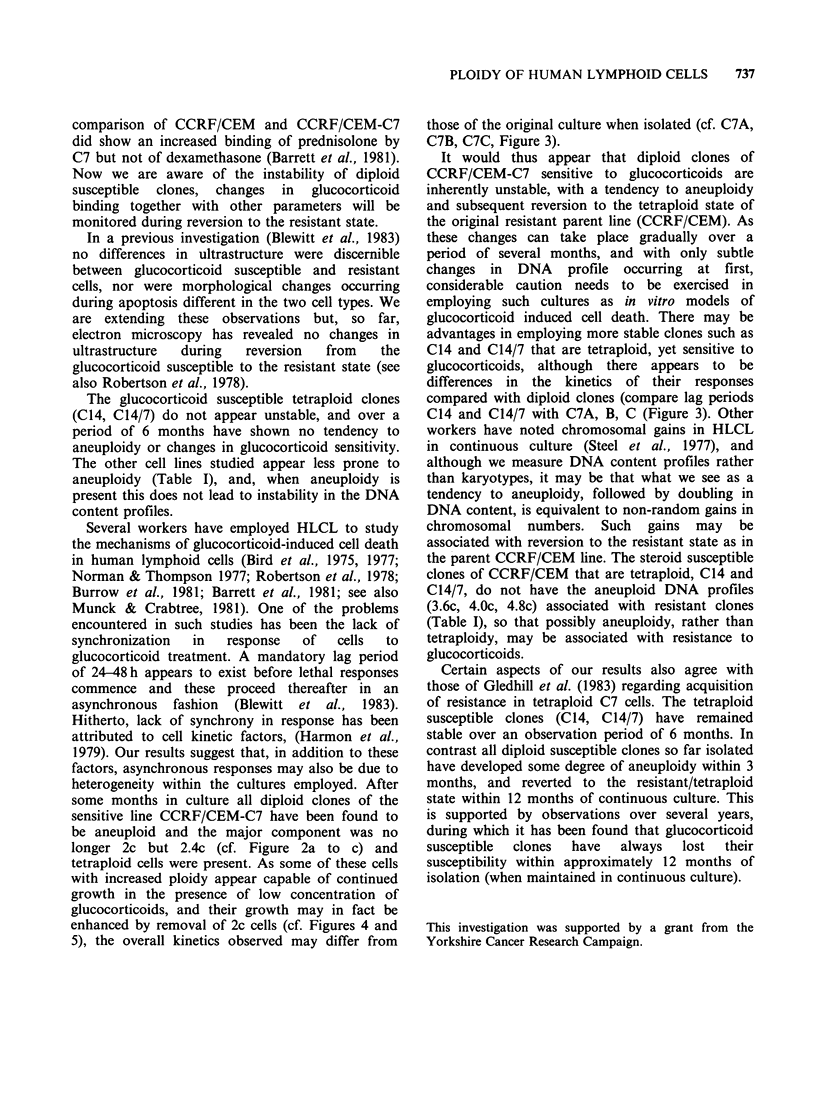

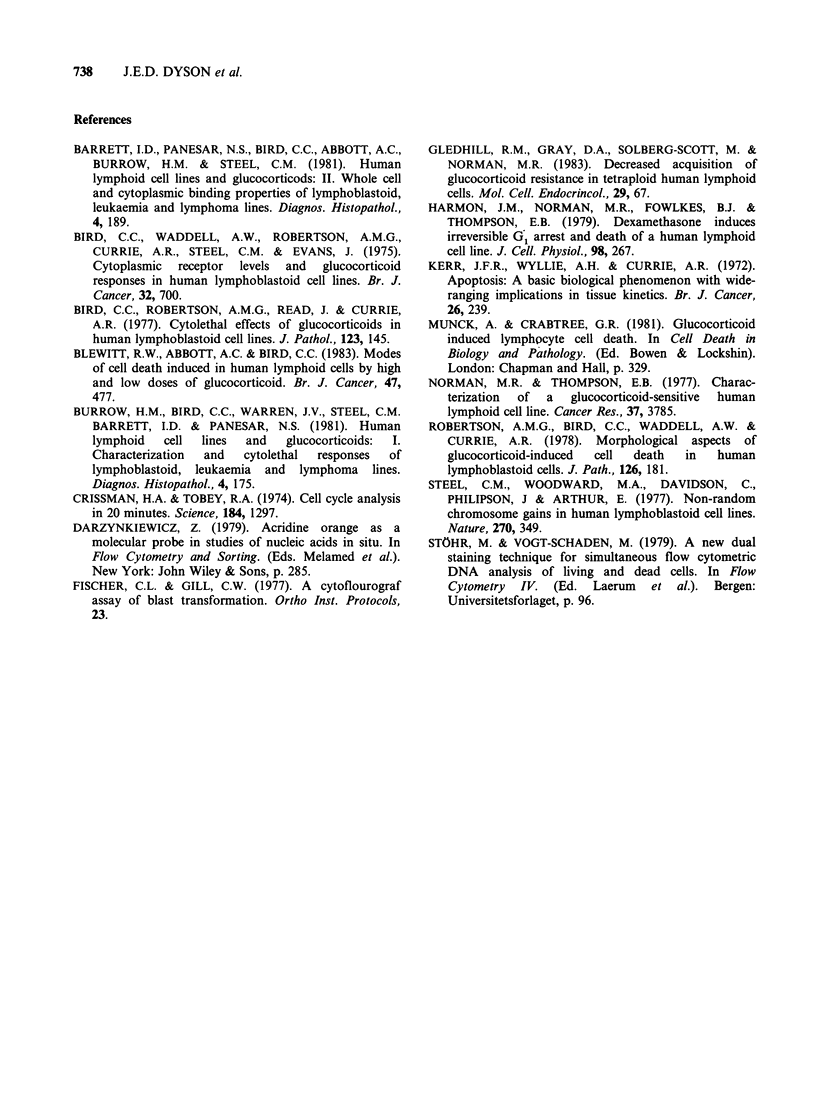


## References

[OCR_00733] Barrett I. D., Panesar N. S., Bird C. C., Abbott A. C., Burrow H. M., Steel C. M. (1981). Human lymphoid cell lines and glucocorticoids: II. Whole cell and cytoplasmic binding properties of lymphoblastoid, leukaemia and lymphoma lines.. Diagn Histopathol.

[OCR_00748] Bird C. C., Robertson A. M., Read J., Currie A. R. (1977). Cytolethal effects of glucocorticoids in human lymphoblastoid cell lines.. J Pathol.

[OCR_00741] Bird C. C., Waddell A. W., Robertson A. M., Currie A. R., Steel C. M., Evans J. (1975). Cytoplasmic receptor levels and glucocorticoid response in human lymphoblastoid cell lines.. Br J Cancer.

[OCR_00753] Blewitt R. W., Abbott A. C., Bird C. C. (1983). Mode of cell death induced in human lymphoid cells by high and low doses of glucocorticoid.. Br J Cancer.

[OCR_00761] Burrow H. M., Bird C. C., Warren J. V., Steel C. M., Barrett I. D., Panesar N. S. (1981). Human lymphoid cell lines and glucocorticoids: I. Characterization and cytolethal responses of lymphoblastoid, leukaemia and lymphoma lines.. Diagn Histopathol.

[OCR_00767] Crissman H. A., Tobey R. A. (1974). Cell-cycle analysis in 20 minutes.. Science.

[OCR_00782] Gledhill R. M., Gray D. A., Solberg-Scott M., Norman M. R. (1983). Decreased acquisition of glucocorticoid resistance in tetraploid human lymphoid cells.. Mol Cell Endocrinol.

[OCR_00788] Harmon J. M., Norman M. R., Fowlkes B. J., Thompson E. B. (1979). Dexamethasone induces irreversible G1 arrest and death of a human lymphoid cell line.. J Cell Physiol.

[OCR_00794] Kerr J. F., Wyllie A. H., Currie A. R. (1972). Apoptosis: a basic biological phenomenon with wide-ranging implications in tissue kinetics.. Br J Cancer.

[OCR_00806] Norman M. R., Thompson E. B. (1977). Characterization of a glucocorticoid-sensitive human lymphoid cell line.. Cancer Res.

[OCR_00811] Robertson A. M., Bird C. C., Waddell A. W., Currie A. R. (1978). Morphological aspects of glucocorticoid-induced cell death in human lymphoblastoid cells.. J Pathol.

[OCR_00817] Steel C. M., Woodward M. A., Davidson C., Philipson J., Arthur E. (1977). Non-random chromosome gains in human lymphoblastoid cell lines.. Nature.

